# Repair of a Syphilitic Aneurysm of the Noncoronary Sinus

**DOI:** 10.1055/s-0040-1714057

**Published:** 2020-11-05

**Authors:** Danielle J. Kinsey, Christopher Zambrano, Alejandro Suarez-Pierre, Aruna Kumar, Elena Isenbergh, Jennifer S. Lawton, Michael P. Siegenthaler

**Affiliations:** 1 Division of Cardiac Surgery, Department of Surgery, Johns Hopkins University School of Medicine, Baltimore, Maryland; 2 Department of Pathology, Suburban Hospital, Johns Hopkins Medicine, Bethesda, Maryland; 3 Department of Infectious Disease, Suburban Hospital, Johns Hopkins Medicine, Bethesda, Maryland; 4 Division of Cardiac Surgery, Suburban Hospital, Johns Hopkins Medicine, Bethesda, Maryland

**Keywords:** aortic root aneurysm, syphilis, sinus of the Valsalva, aortitis

## Abstract

About one-tenth of patients with untreated chronic syphilis and tertiary syphilis develop structural complications involving the coronary ostia, ascending aorta, or aortic root. We describe a unique case of a large aortic root aneurysm of the noncoronary sinus with extrinsic compression of the right coronary artery, a complication of tertiary syphilis. Surgical intervention involved valve-sparing aortic root reconstruction with right coronary ostia reimplantation (hemi-Yacoub). The patient's postoperative course was uneventful; he is healthy approximately 2 years later.

## Introduction

Patients with untreated syphilis may progress from latent infection to tertiary syphilis in a period between 10 and 40 years, 1 a process that can be accelerated by coinfection with HIV. 2 Approximately 70 to 80% of patients with untreated tertiary syphilis will experience chronic aortitis, which is often asymptomatic but may result in any of the following significant cardiovascular complications if not addressed: aortic valve compromise, coronary ostial stenosis, or aortic aneurysm. Decades after the initial infection, 10% of patients with untreated syphilitic aortitis will progress to severe cardiovascular disease, which could be avoided using penicillin as treatment. 3 Usually, aneurysms occur proximal to the left subclavian, given that these spirochetes target the vasa vasorum, which exists predominately in the ascending aorta due to lymphatic supply. Syphilitic aneurysms infrequently are isolated to the aortic root, with the rarest form being confined to the noncoronary sinus.

## Case Presentation

**Fig. 1:**
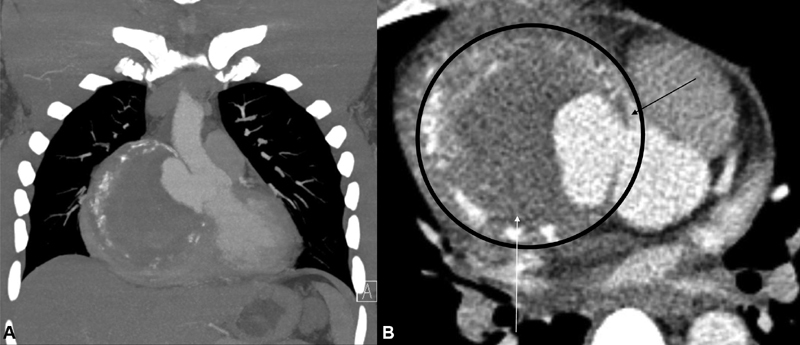
Computed tomography angiography of the chest demonstrating a 10 cm aneurysm of the non-coronary sinus with laminated calcifications. ( **A** ) Coronal section with intra-arterial contrast demonstrating partial filling of the aneurysmal sac. ( **B** ) Axial section of the aortic root with the aneurysmal sac (encircled) compressing the proximal right coronary artery (black arrow) and the right atrium (white arrow).

**Fig. 2:**
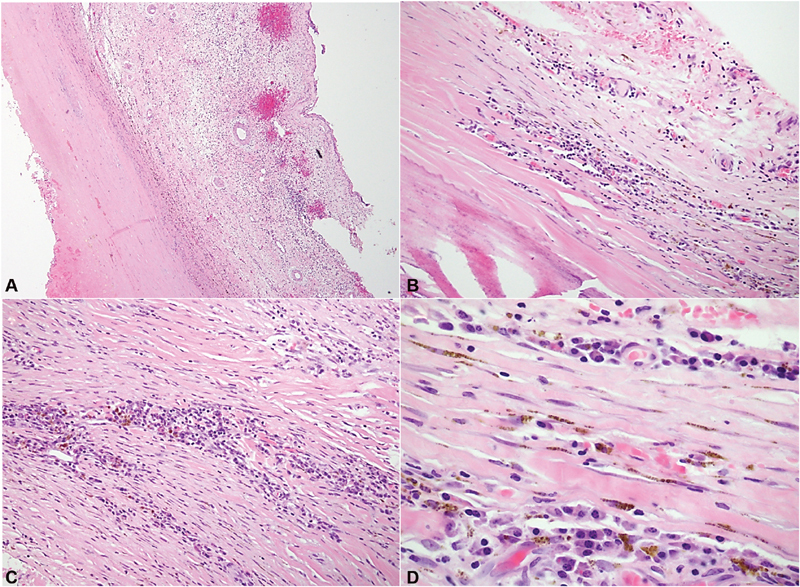
Microscopic examination revealed thickened fibrous walls with chronic inflammatory cells, mostly plasma cells. No granulomas are seen. ( **A** ) H&E stain at ×2 magnification showing full thickness fibrous architecture. The aneurysm included all layers of the aorta, showing it is a true aneurysm. ( **B** and **C** ) H&E stain at ×10 magnification. Muscle with chronic inflammatory cells. ( **D** ) H&E at ×40 magnification demonstrating plasma cells. Abbreviations: H&E, hematoxylin and eosin stain.

A 44-year-old male presented to the emergency department with new onset of severe midsternal chest pain exacerbated during deep inspiration. The patient had no past medical nor family history of cardiovascular disease. He had a history of syphilis confirmed through fluorescent treponemal antibody absorption and a leg wound 5 years prior that was reported as pyogenic granuloma upon excisional biopsy. Although testing for HIV was negative, the patient was taking antiretroviral medication for prophylaxis. Cuff blood pressures were 150/90 mm Hg on both upper limbs with normal distal pulses on all limbs. There were no signs of end organ malperfusion or altered mental status. An electrocardiogram (ECG) was notable for sinus tachycardia and 2-mm ST segment elevation on leads II, III, and aVF (augmented vector foot). Blood work indicated normal troponin levels, an elevated D-dimer, prothrombin time of 11.4 seconds, and an international normalized ratio of 1.07. A computed tomography angiography demonstrated a 9.6 cm × 9.5 cm × 10.4 cm aneurysm of the noncoronary sinus with laminated calcifications ( Fig. 1A ), causing extrinsic compression of the proximal right coronary artery ( Fig. 1B ) and displacement of the right atrium inferiorly and laterally. The patient was emergently taken to the operating room, arterial cannulation was obtained through a chimney graft sewn onto the right axillary artery, and venous cannulation through the right common femoral vein prior to sternotomy. Approximately 50 mL of dense serous fluid was collected from the pericardial space, which demonstrated abundant neutrophils without the presence of bacterial growth on culture. Cross clamp and antegrade cardioplegia were established. Approximately 300 mL of thrombus was obtained from the aneurysmal sac. An aortic repair was performed with a Valsalva's graft replacing the noncoronary sinus. A felt strip was sewn to the aortic root at the level of the aortic valve annulus after complete excision and debridement of the remaining coronary sinus tissues with subsequent reimplantation of the right coronary artery (hemi-Yacoub). This was performed due to involvement of the proximal ascending aorta. The patient was kept in Trendelenburg's position on low flow axillary partial cardiopulmonary bypass for 10 minutes while the heart was ejecting to direct any potential debris toward the lower body, given the substantial stroke risk secondary to dislodgement of thrombus during aneurysmal debridement. Coming off bypass, transesophageal echocardiography confirmed normal aortic valve function with trace central aortic insufficiency. The procedure required 186 minutes of cardiopulmonary bypass and 136 minutes of aortic cross-clamp. The patient was extubated 6.5 hours after the conclusion of the operation and experienced postoperative atrial fibrillation amenable to rhythm control. He completed a 2-week course of intravenous penicillin G (4,000,000 UI in every 4 hours) and was discharged 15 days after surgery. Microscopic examination of the resected aortic tissue exhibited hypercellularity without intramural granulomas or necrosis ( Fig. 2 ). No organisms were observed on histological examination of the specimen. The patient continues to do well almost 2 years after the operation.

## Discussion

This case report describes an isolated noncoronary sinus of Valsalva's aortic aneurysm that occurred secondary to syphilis infection, which was surgically repaired. The size of the patient's aneurysm was restricting flow through the right coronary that resulted in ECG changes consistent with inferior wall ischemia. The large aneurysm produced right atrial and ventricular compression and aortic valve insufficiency from aortic annular dilatation. The right coronary artery was found to be patent and was successfully reimplanted into the Valsalva's graft. Definitive diagnosis of syphilitic aneurysm was made with the fluorescent treponemal antibody absorption test (sensitivity, 100%; specificity, 96%) 4 and histological confirmation of changes consistent with syphilitic aortitis ( Fig. 2 ).

Syphilitic aneurysms occur through the progression of endarteritis obliterans, necrosis of the tunica media, chronic inflammation, and fibrosis during a period of infection without signs of disease that would warrant further investigation. 3 5 These mechanisms make the aorta inelastic, dilated, and weakened, and ultimately lead to the formation of a saccular aneurysm, with unilateral extension from one side of the vessel.

The widespread access to penicillin in developed countries makes the presentation of syphilitic cardiovascular complications relatively uncommon, with most case reports coming from developing nations. Definitive diagnosis is rare because various etiologies of aortitis have similar presentations and the exact cause of disease is often unclear. The few cases reported progress to the point of severe clinical deterioration, requiring replacement surgery or are diagnosed only at autopsy. If rupture of a syphilitic aneurysm occurs, patients may experience symptoms ranging from chest pain and dyspnea to aortic insufficiency, heart failure, and shock. 6 7 Some patients have disease characterized by intimal plaques and scarring called “tree-bark aorta,” 6 while others are found to have an aneurysm that extends beyond the aorta into the brachiocephalic artery. 7 When intervention is unsuccessful or not timely, diagnosis is only made at the time of autopsy. 8

Since this aneurysm was surgically repaired and postoperative treatment for syphilis was monitored until completion, the patient is doing well at approximately 2 years. Close follow-up will continue to occur due to the possibility for recurrence in cases of *Treponema pallidum* infection.
